# Solvent-Resistant UV-Cured Polysulfone Support Membranes Using a Green Solvent

**DOI:** 10.3390/membranes12010001

**Published:** 2021-12-22

**Authors:** Angela Dedvukaj, Peter Van den Mooter, Ivo F. J. Vankelecom

**Affiliations:** Membrane Technology Group (MTG), Division cMACS, Department of Microbial and Molecular Systems (M^2^S), Faculty of Bioscience Engineering, KU Leuven, Celestijnenlaan 200F, P.O. Box 2454, 3001 Leuven, Belgium; angela.dedvukaj@kuleuven.be (A.D.); peter.vandenmooter@kuleuven.be (P.V.d.M.)

**Keywords:** green solvent, Tamisolve^®^ NxG, UV-curing, solvent-resistant support, polysulfone, phase inversion

## Abstract

Solvent-resistant UV-cured supports consisting of a semi-interpenetrating network of polysulfone (PSf) and cross-linked poly-acrylate were successfully synthesized for the first time using an alternative, non-reprotoxic, and biodegradable solvent. Tamisolve^®^ NxG is a high-boiling, dipolar aprotic solvent with solubility parameters similar to those of dimethylformamide (DMF) and N-methyl-2-pyrrolidone (NMP), making it an eco-friendly alternative. The support membranes, prepared via UV-curing followed by non-solvent-induced phase inversion, can serve as a universal solvent-resistant support for the synthesis of a broad set of membranes, for which the selective layer can be deposited from any solvent. Parameters such as UV irradiation time and intensity, as well as the concentrations of PSf, penta-acrylate, and photo-initiator in the casting solution were varied to obtain such supports. The characteristics of the resulting supports were investigated in terms of separation performance, hydrophobicity, porosity, degree of acrylate conversion, and pure water flux. The resulting membranes showed improved chemical resistance in solvents such as ethyl acetate, NMP, tetrahydrofuran (THF), and toluene. Solvent-resistant supports with different pore sizes were synthesized and used for the preparation of thin film composite (TFC) membranes to demonstrate their potential. Promising separation performances with Rose Bengal (RB) rejections up to 98% and water permeances up to 1.5 L m^−2^ h^−1^ bar^−1^ were reached with these TFC-membranes carrying a polyamide top layer synthesized via interfacial polymerization.

## 1. Introduction

Driven by the increased focus on sustainability and health, membrane technology has gained interest and is used in various applications as a more environmentally friendly separation technique due to its better energy efficiency, less waste production, and lower capital and operation costs compared to conventional separation processes [1,2,3,4,5,6,7,8]. Polymeric membranes are widely used due to their good processability and low cost [9,10]. PSf is a commercial polymer with excellent thermal and mechanical properties and is, therefore, used in a wide scope of membrane applications [11,12]. Common solvents for PSf and other commercial polymers are N-methyl-2-pyrrolidone (NMP), *N*,*N*-dimethylformamide (DMF) and *N*,*N*-dimethylacetamide (DMAc) [12]. However, these solvents are harmful and toxic and may cause serious long-term problems for human health and the environment [12,13]. The Green Chemistry principles encourage the substitution of harmful compounds with safer ones; therefore, Tamisolve^®^ NxG was used in this study as an alternative, non-reprotoxic, and biodegradable solvent [13]. Tamisolve^®^ NxG is a high-boiling, dipolar aprotic solvent with similar solubility parameters to those of DMF and NMP, which make it an eco-friendly alternative solvent for membrane synthesis [12,13]. The membrane synthesis of polymeric membranes often occurs through non-solvent-induced phase separation (NIPS) in which the cast polymer film is immersed in a non-solvent bath where the casting solution demixes and the polymer precipitates [11,14,15,16,17,18]. Although PSf possesses favorable properties such as good mechanical properties and thermal stability, it remains soluble in many aprotic solvents, which limits its use in solvent-based applications [12,13,19,20]. Not only during its actual application but also during membrane cleaning or during synthesis of more selective membrane layers when used as support, the chemical stability of the material is crucial [20]. Therefore, further modification of the via NIPS-synthesized PSf membranes is needed to increase the solvent resistance [19,20]. Chemical cross-linking is commonly performed to increase the chemical resistance of membranes. The chemical cross-linking of a polyimide (PI) membrane with diamine is a well-known example. However, it requires extra synthesis steps, including use of toxic compounds, and is less convenient for PSf due to the absence of groups on this polymer that can react easily [11,19,20,21]. High-temperature electron-beam irradiation can be applied for cross-linking of chemically less reactive polymers, but the equipment is expensive and the high temperatures can damage the membrane structure. Instead, UV curing was implemented in this study, which is a simple and versatile method to cross-link PSf membranes [11,22]. By addition of a cross-linker (XL) and a photo-initiator (PhIn) to the polymer solution and a subsequent UV irradiation after NIPS, a semi-interpenetrating network of PSf with the reached XL could be formed. This network is known to increase the solvent stability properties of the synthesized membrane [22,23]. In this study, the influence of UV irradiation and the effect of PSf, XL (pentaerythritol penta-acrylate), and PhIn (trimethylbenzoyl diphenylphosphine oxide) on the filtration performance, solvent resistance, and morphology of the UV-cured PSf membrane were studied to optimize their use as support. The obtained UV-cured PSf support served as a universal solvent-resistant support for the synthesis of a broad set of membranes requiring organic solvents during further preparation of the selective layer.

## 2. Materials and Methods

### 2.1. Materials

Polysulfone 3010 (PSf, Ultrason^®^ S 3010, Mw = 37–45 k) and polysulfone 6010 (PSf Ultrason^®^ S 6010, Mw = 45–55 k) were obtained from BASF SE (Ludwigshafen, Germany). PSf was dried for at least 24 h at 100 °C prior to use and was then dissolved in TamiSolve^®^ NxG (Taminco, Gent, Belgium) together with 2,4,6-trimethylbenzoyl diphenylphosphine oxide (TPO, Darocur^TM^) and pentaerythritol penta-acrylate (SR399LV, Sartomer^TM^). Trimesoyl chloride (TMC, 98%), m-phenylenediamine (MPD, +99%), heptane (+99%), sodium dodecyl sulfate (SDS, 99%), and 1-methyl-2-pyrrolidone (NMP, 99%) were bought from Acros Organics (Geel, Belgium). Triethylamine (TEA), Rose Bengal (RB), toluene (99.8%), ethyl acetate (EtOAc, anhydrous, 99.8%), and tetrahydrofuran (THF, 99.9%) were purchased from Sigma-Aldrich (Diegem, Belgium).

### 2.2. Membrane Synthesis

#### 2.2.1. Preparation of PSf-Based Supports

Solutions of PSf in TamiSolve were prepared with different PSf concentrations (10–20 wt%) and stirred at 80 °C for 7 h. After cooling down, the photo-initiator TPO (0.3 or 1 wt%) and the cross-linker SR399LV (0.5, 0.75, 1.5, 1.25, or 2 wt%) were added in the dark, and the solution, covered by aluminum foil, was stirred further for an additional 2 h and left to degas overnight. A 250-µm-thick film of these polymer solutions was cast on a PE/PP non-woven substrate (Novatex 2471), which was impregnated with TamiSolve, at a speed of 1.29 m min^−1^ using an automated casting knife (Porometer, Nazareth, Belgium). The liquid film was then transferred to a deionized water-containing coagulation bath to perform NIPS. Both casting and NIPS were performed in the dark.

#### 2.2.2. UV Curing of PSf-Based Support Membranes

After synthesis of the PSf-based support membranes, cross-linking was performed via UV curing using the UV LED curing system Semray^®^-UV4003 LED (UVio Ltd., Thatcham, UK) at a peak wavelength of 365 nm. An energy density of 14 or 28 J cm^−2^ was used to cure the supports for 10 s at a distance of 36 mm.

#### 2.2.3. Synthesis of PA TFC Membranes

PA TFC membranes were obtained by interfacial polymerization. The UV-cured support membranes were immersed in an aqueous solution containing 2% (*w*/*v*) MPD, 2% (*w*/*v*) TEA, and 0.1% (*w*/*v*) SDS for 30 min. Excess solution was removed with a rubber wiper. A 0.1% (*w*/*v*) TMC in heptane solution was then poured on top of the support surface and left to perform IP for 1 min. The surface was rinsed with heptane to remove the unreacted TMC and dried in air for 2 min. The TFC membranes were stored in DI water until further use [24].

### 2.3. Membrane Characterization

#### 2.3.1. Filtration Experiments

The filtration experiments were carried out in a high-throughput (HT) membrane filtration module, which allowed a simultaneous filtration of 16 membrane coupons, each with an active area of 1.54 × 10^−4^ m^2^ [25]. The pressure-driven dead-end filtrations were performed at room temperature and pressures ranging from 2 to 20 bar while stirring the feed solution at 350 rpm to minimize the effect of concentration polarization. The feed existed of 17 µM RB in Milli-Q water. For each membrane, three to 4ffour coupons were tested from which the performance was averaged and the standard deviation was taken.

The permeance (*L_p_*) is the ratio of the amount of collected permeate (*V*, L) to the active surface area of the membrane (*A*, m^2^), collection time (*t*, h), and applied pressure (*p*, bar). and was calculated using the following equation:

(1) $$L_{p}=\frac{V}{At\Delta P}\;(L\;m^{-2}\;h^{-1}\;{\mathrm{bar}}^{-1})$$

The rejection of RB was calculated using:

(2) $$R=\left(1-\frac{c_{p}}{c_{f}}\right)\times 100\;\left(\%\right)$$

with *c_f_* as the RB concentration in the feed and *c_p_* as the RB concentration in the permeate.

The concentration of RB was determined by UV-Vis spectrophotometry at λ_max_ = 550 nm (Shimadzu UV-1800 UV/Visible Scanning Spectrophotometer).

#### 2.3.2. Contact Angle Measurements

The hydrophobicity of the UV-cured PSf-based supports was measured using a Drop Shape Analysis System DSA 100 (Krüss, Matthews, NC, USA). The sessile drop method was performed. The contact angle was measured eight times for each support and an average value and standard deviation were taken.

#### 2.3.3. Attenuated Total Reflectance Fourier Transform Infrared Spectroscopy (ATR-FTIR)

Attenuated total reflectance Fourier transform infrared (ATR-FTIR) spectroscopy was used to determine the conversion of the acrylate double bonds. A Bruker Vertex 70 FTIR spectrometer with a diamond crystal was used. The measurements were performed on the top surface of the support membranes taking 64 scans at a resolution of 4 cm^−1^ at wavenumbers between 4000 and 650 cm^−1^. For each membrane, four ATR-FTIR spectra were taken of four different spots on the membrane and an average value and standard deviation were taken from the absorbance peaks. The absorbance peaks at 810 cm^−1^ and 1728 cm^−1^, which corresponded to the *C=C* group and *C=O* group, respectively, were used to calculate the conversion with following equation:

(3) $$Conversion=\left(1-\frac{{\left(\frac{C=C}{C=O}\right)}_{\text{cured}}}{(\frac{C=C}{C=O}{)}_{\text{non-cured}}}\right)\times 100\;\left(\%\right)$$

#### 2.3.4. Scanning Electron Microscopy (SEM)

Scanning electron microscopy (SEM, JEOL-JSM-6010LV) was used to analyse the morphology of the top-layer surface of the TFC membranes. Before measurement, the samples were first coated with a conductive gold/palladium layer with a JEOL JFC-1300 Auto Fine Coater.

#### 2.3.5. Swelling/Solvent Resistance Test

For the solvent-resistance test, small pieces of the PSf membranes (which were cast on a glass plate without non-woven support) were immersed in various solvents for at least 48 h. The solvents used for this test were ethyl acetate, NMP, THF, and toluene.

#### 2.3.6. Porosity Factor

A factor including both porosity and pore radius, the porosity factor of *εr_p_^2^*, was calculated based on the Hagen–Poiseuille pore flow model:

(4) $$L_{p}=\frac{\varepsilon r_{p}^{2}}{8\mu \delta_{m}}\;(L\;m^{-2}\;h^{-1}\;{\mathrm{bar}}^{-1})$$

where *L_p_* is the membrane permeance (m^2^/(m Pa s)), ε is the membrane porosity, *r_p_* is the pore radius (m), *µ* is the solution viscosity (Pa s), and *δ_m_* is the membrane thickness (m). An increasing porosity factor can indicate a higher porosity, higher pore radius, or both.

## 3. Results and Discussion

### 3.1. Effect of PSf Type and Photo-Initiator on Membrane Performance and Solvent Stability

**Figure 1:**
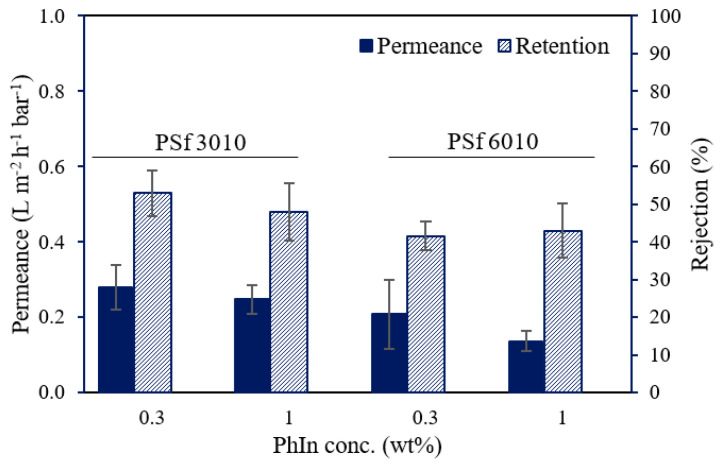
Water permeance and RB rejection of UV-cured PSf (10 wt%) membranes synthesized with 5 wt% XL, different PSf types (PSf Ultrason^®^ S 3010 or 6010), and different PhIn concentrations (0.3 or 1 wt%).

As a preliminary test, UV-cured membranes with 10 wt% PSf, 5 wt% XL, and 0.3 or 1 wt% PhIn were synthesized. The performance for both types of PSf (Ultrason^®^ S 3010 and Ultrason^®^ S 6010) is shown in Figure 1. As expected, the supports synthesized with PSf 6010 (Mw = 45–55 k) showed lower permeances compared to PSf 3010 (Mw = 37–45 k). The viscosity of the casting solution increased with increasing molecular weight (MW), which resulted in a denser membrane caused by a more delayed demixing during the phase inversion [19,26,27]. Increasing the PhIn concentration did not have a significant effect on the performance of the UV-cured supports. It is suggested that a concentration of 0.3 wt% PhIn produced a sufficient amount of radicals to initiate the photo-induced free-radical polymerization reaction.

To demonstrate the effect of UV curing, PSf type, and photo-initiator concentration on the solvent stability, the membranes were immersed in ethyl acetate, NMP, THF, and toluene. M0 is the reference membrane, which was not UV-cured, and M1–M4 are the UV-cured supports. The compositions of the membranes are shown in Table 1.

A qualitative observation of the solvent stability of the reference membranes and the UV-cured PSf membranes can be found in Table 2. UV curing improved the membrane stability significantly. The reference membrane (M0) swelled in ethyl acetate while it was not stable in all other solvents. The UV-cured PSf membranes were stable in ethyl acetate and toluene and swelled in NMP and THF. Since all UV-cured PSf membranes (M1–M4) showed similar solvent stability, the polymer type and PhIn concentration had no significant effect on the solvent stability. Again, a lower PhIn concentration was sufficient to initiate cross-linking; hence, 0.3 wt% PhIn was used in further experiments.

### 3.2. Effect of Cross-Linker Concentration in the Casting Solution

**Figure 2:**
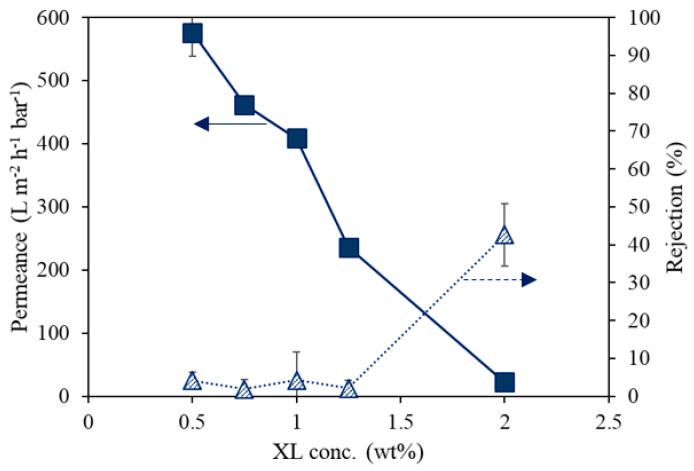
The effect of cross-linker on the performance of UV-cured PSf membranes consisting of 10 wt% PSf and 0.3 wt% PhIn.

Figure 2 presents the effect of cross-linker concentration in the casting solution on water permeance and RB rejection of the UV-cured PSf supports. The PSf and PhIn concentration were kept constant at 10 wt% and 0.3 wt%, respectively. With increasing cross-linker concentration (0.5 to 2 wt%), the permeance decreased (575.9 to 22.5 L m^−2^ h^−1^ bar^−1^) and the rejection increased (4.2 to 42.7%). Increasing the cross-linker concentration led to a densification of the membrane. The cross-linker also acted as an additive in the casting solution, which increased the viscosity of the casting solution. This can result in a denser membrane caused by the delayed demixing effect during phase inversion [21,26,27].

In Table 3, the solvent stability of the UV-cured PSf supports prepared with different cross-linker concentration is presented. UV-cured PSf supports with cross-linker concentrations of 0.5 to 1.5 wt% swelled in ethyl acetate and dissolved in NMP, THF, and toluene. Hence, these membranes did not show improved solvent stability compared to the reference membrane (no cross-linker). The UV-cured PSf supports containing 2 wt% cross-linker showed improved solvent stability. The membrane became stable in ethyl acetate and toluene and swelled in NMP and THF. Thus, a minimum of 2 wt% cross-linker was required to obtain sufficient cross-linking and improved solvent stability.

**Figure 3:**
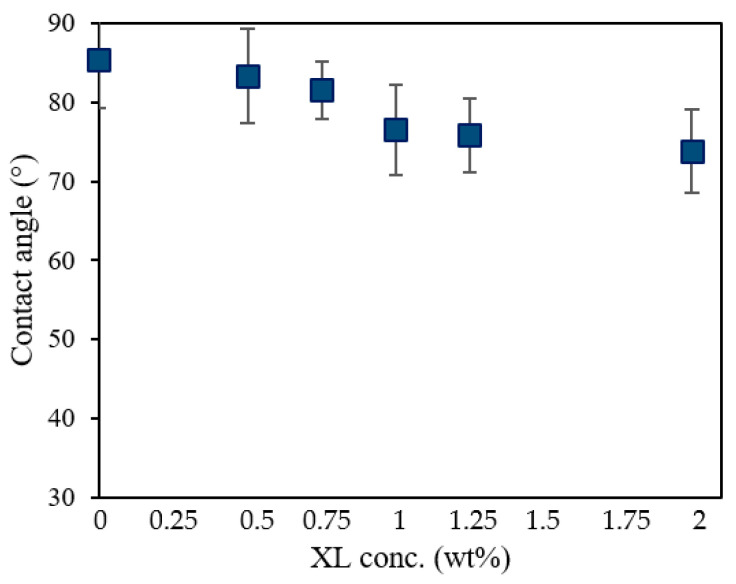
The contact angle of UV-cured PSf membranes consisting of 10 wt% PSf and 0.3 wt% PhIn with different cross-linker concentrations.

Figure 3 shows the contact angle of UV-cured PSf membranes consisting of 10 wt% PSf and 0.3 wt% PhIn with different cross-linker concentrations. Increasing the concentration of the cross-linker led to decreasing contact angles, and, hence, a less hydrophobic membrane, due to the hydrophilic character of the cross-linker.

**Figure 4:**
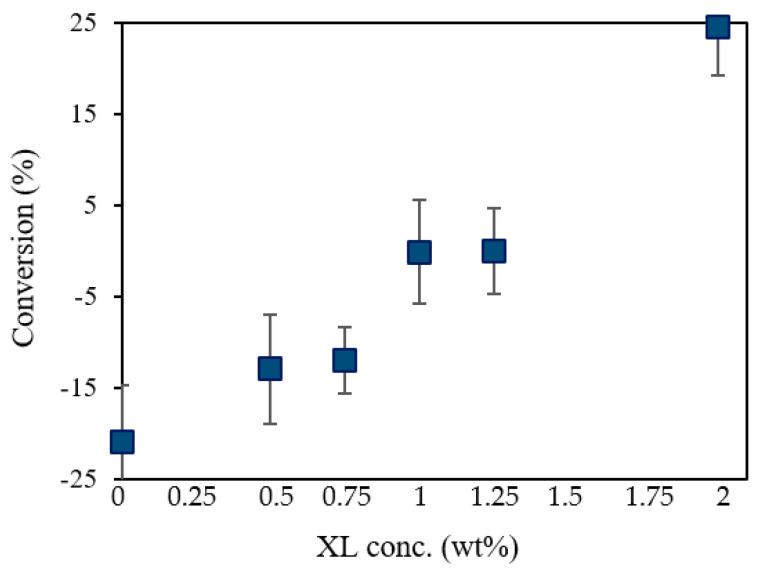
Cross-linker conversion of UV-cured PSf membranes consisting of 10 wt% PSf, different cross-linker concentrations, and 0.3 wt% PhIn.

The degree of cross-linker conversion of UV-cured PSf supports prepared with 10 wt% PSf, different cross-linker concentrations, and 0.3 wt% PhIn is shown in Figure 4. Higher cross-linker concentration led to a higher degree of acrylate conversion. With increasing XL concentration at constant PhIn concentration, the probability for PhIn radicals to react with XL monomers, and thus potentially polymerize, increased, explaining the higher degree of acrylate conversion [22]. Note that the negative values can be due to the difficulty in correctly determining the baseline. Only a trend can thus be taken from this graph.

### 3.3. Influence of Energy Density

**Figure 5:**
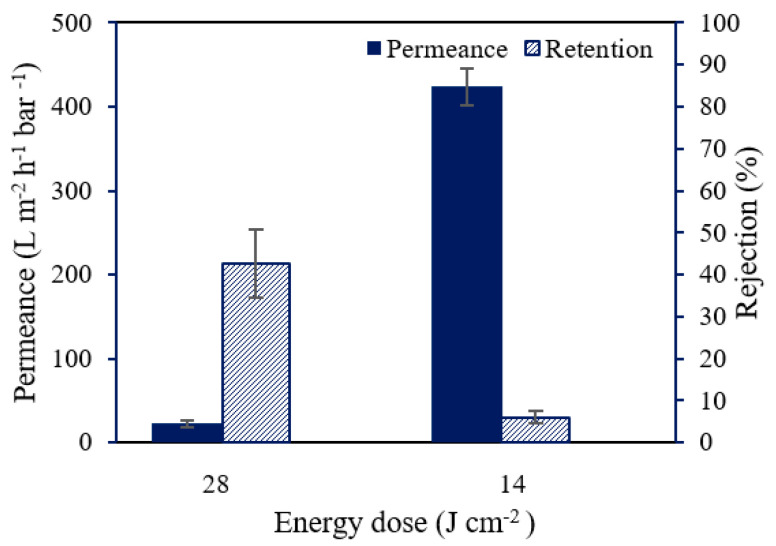
The effect of energy dose on the performance of the UV-cured PSf supports. Both membranes were prepared from a solution containing 10 wt% PSf, 2 wt% XL, and 0.3 wt% PhIn.

In the abovementioned experiments, the energy dose to cure the membranes was 28 J cm^−2^ with an irradiation time of 10 s. In Figure 5, the effect of 50% reduction in energy dose is shown. When the energy density was halved, the permeance of the UV-cured PSf membranes increased drastically from 22.5 to 406.3 L m^−2^ h^−1^ bar^−1^ while the RB rejection decreased from 42.7 to 7.7%. A higher energy dose can result in a higher cross-linking degree, which can explain the higher rejection for the membrane cured with the highest energy density [23]. On the other hand, as could be clearly observed experimentally, a high-energy dose generated more heat at the membrane surface. This can cause drying of the pores on the membrane surface and, therefore, some pore collapse with significant loss of surface porosity [28]. The decreased porosity and pore sizes led to an increased rejection and decreased permeance [29]. To limit pore collapse, 14 J cm^−2^ was applied in following research.

### 3.4. Influence of Cross-Linker on Supports Cast with Different PSf Concentration

**Figure 6:**
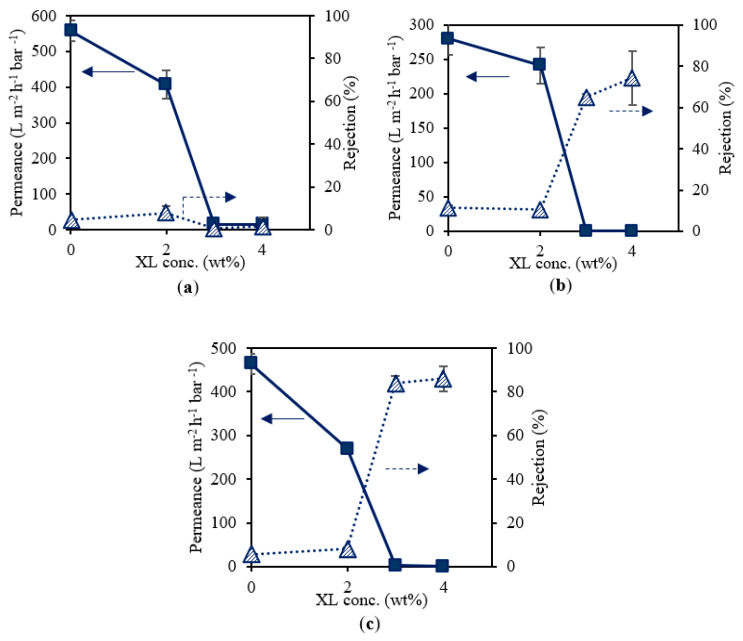
Water permeance and RB rejection of membranes consisting of 10 wt% (**a**), 12 wt% (**b**), and 14 wt% (**c**) PSf, 0.3 wt% PhIn, and varying cross-linker (XL) concentrations.

Figure 6 presents the effect of cross-linker on the water permeance and RB rejection of a set of three UV-cured PSf supports cast from different PSf concentrations (10, 12, and 14 wt% PSf). To obtain sufficient cross-linking, 2, 3, and 4 wt% cross-linkers were used and a reference membrane containing no cross-linker was included. Figure 6a shows the performance of 10 wt% PSf membranes for various cross-linker concentrations. A decrease in permeance can be observed when increasing the cross-linker concentration. No clear trend could be found for the RB rejection, which remained very low. Figure 6b,c presents supports cast from 12 and 14 wt% PSf solutions, respectively. For both types, similar trends in performance for increasing cross-linker concentration were observed. As mentioned above, increasing the cross-linker concentration led to a more cross-linked network, and an increased viscosity of the casting solution induced delayed demixing during phase inversion. Together with the changed hydrophobicity, this led to a denser membrane with lower permeance and higher rejection. However, as can be seen on all graphs in Figure 6, cross-linker concentrations of 3 wt% or more resulted in a significant permeance drop to 0.2–2 L m^−2^ h^−1^ bar^−1^. In order to obtain suitable supports with low mass transfer resistance, 2 wt% cross-linker was further used. Additionally, from Table 4, all UV-cured PSf membranes consisting of 2 wt% cross-linker or above showed an improved solvent stability compared to the reference membranes without cross-linker. The same results were obtained for 10, 12, and 14 wt% PSf supports.

### 3.5. Porosity Factor

**Figure 7:**
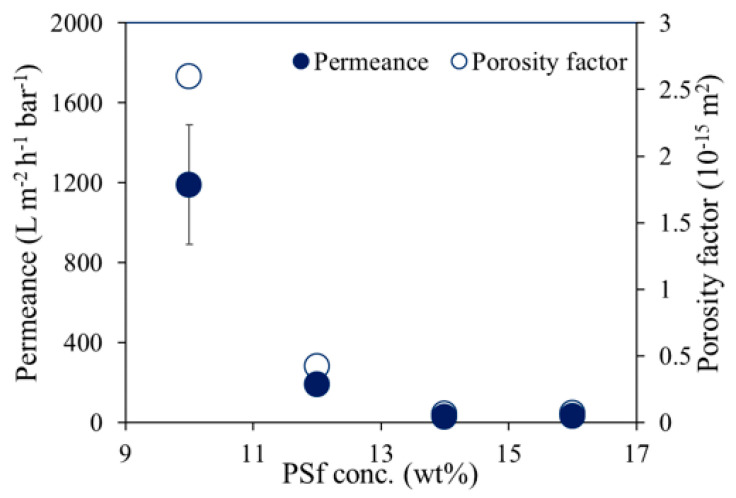
Water permeance and the porosity factors of the UV-cured PSf supports that were prepared using different PSf concentrations, 2 wt% XL, and 0.3 wt% PhIn.

In Figure 7, the water permeances and the porosity factors of the UV-cured PSf supports are shown. These membranes were prepared using different PSf concentrations, 2 wt% cross-linker, and 0.3 wt% photo-initiator. As expected, both permeance and porosity factor decreased with increasing PSf concentration. A higher PSf concentration at the polymer/non-solvent interface when the membrane was immersed in the coagulation bath during NIPS slowed down the in-diffusion of the non-solvent, which resulted in denser skin layers with lower permeances [29,30]. Additionally, all resulting UV-cured PSf supports showed improved solvent stability when immersed in various solvents.

### 3.6. Performance of TFC Membranes Using UV-Cured PSf Supports

**Figure 8:**
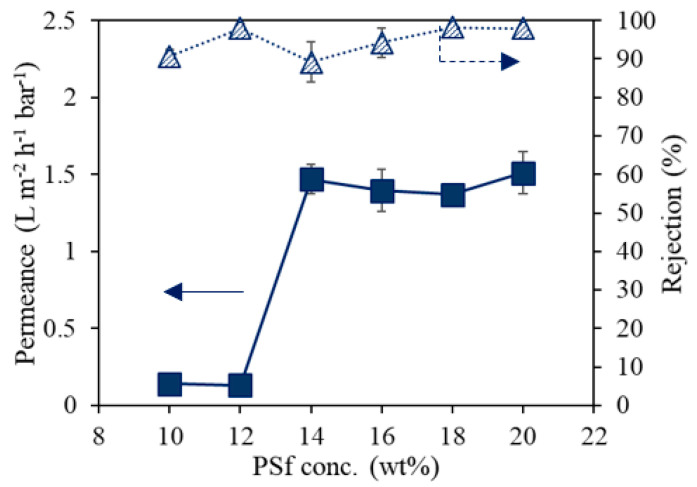
Water permeance and RB rejection of PA TFC membranes synthesized on UV-cured PSf supports with 2 wt% cross-linker, 0.3 wt% photo-initiator, and various initial PSf concentrations.

**Figure 9:**
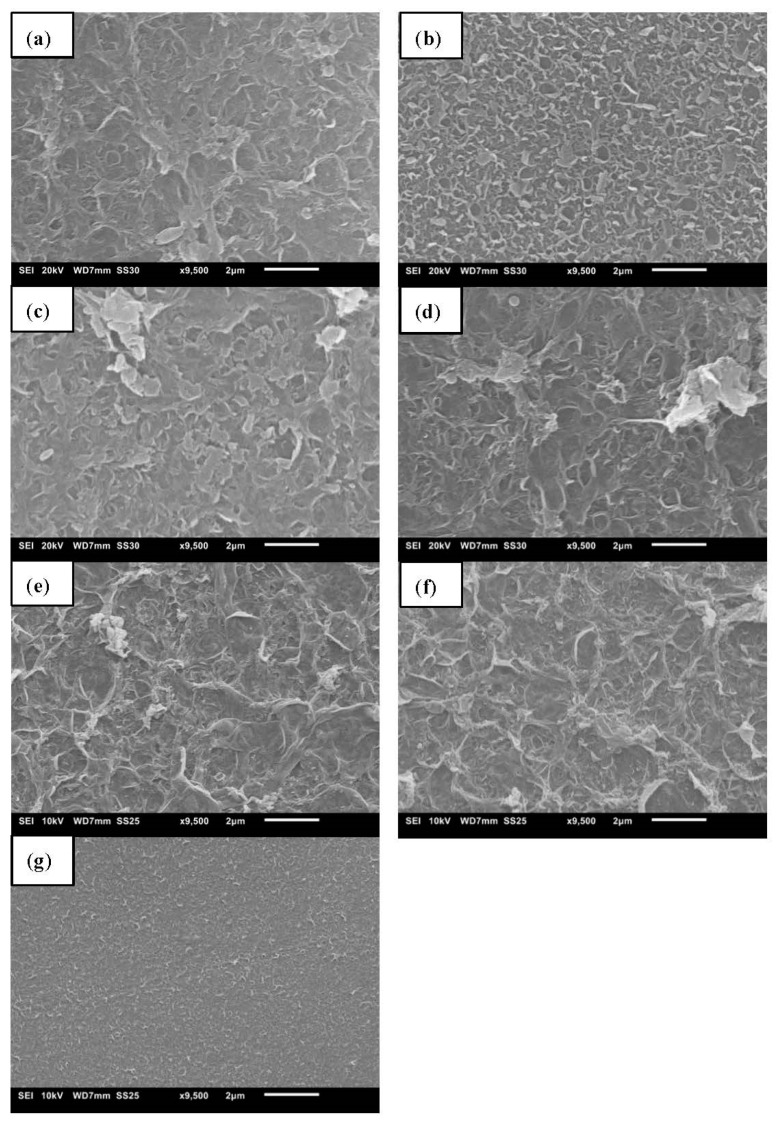
SEM surface images of PA TFC membranes synthesized on UV-cured PSf supports with initial PSf concentrations of (**a**) 10, (**b**) 12, (**c**) 14, (**d**) 16, (**e**) 18, (**f**) 20, and (**g**) 22 wt%.

PA TFC membranes were synthesized on various UV-cured PSf supports prepared with 2 wt% cross-linker, 0.3 wt% photo-initiator, and different PSf concentrations. The water permeances and RB rejections of the prepared PA TFC membranes are shown in Figure 8. The lower permeance of the PA TFC membranes prepared with 10 and 12 wt% PSf was opposite to what was expected, namely, a more open support that would lead to a higher permeance of the TFC membranes [31]. However, the supports with 10 and 12 wt% PSf had a higher XL/PSf ratio and were, hence, less hydrophobic. The pores of the support can, therefore, be wetted more easily and the reaction of TMC with MPD to form PA will be influenced substantially [32]. The permeance of the TFC membranes prepared with 14–20 wt% PSf had similar water permeance while the rejections increased from 89 to 98%. The higher rejection was probably due to the lower porosity of membranes with higher PSf concentration, which resulted in a better formation of PA at the support surface, decreasing the chances for defects [29]. SEM surface images of the TFC membranes prepared on different UV-cured PSf supports are shown in Figure 9. All SEM surface images showed a ridge and valley morphology, which is typical for a PA top layer [31]. Hence, the SEM images confirmed successful polymerization. Overall, PA TFC membranes with good performance were successfully synthesized, which demonstrated the potential of UV-cured PSf membranes as solvent-resistant supports for the preparation of TFC membranes.

## 4. Conclusions

UV-cured supports consisting of a semi-interpenetrating network of PSf and cross-linked poly-acrylate were successfully synthesized using Tamisolve^®^ NxG as an alternative, non-reprotoxic, and biodegradable solvent. UV curing improved the membrane stability significantly in solvents such as ethyl acetate, NMP, THF, and toluene. During phase inversion, the cross-linker acted as an additive in the casting solution, which resulted in a denser membrane. Increasing the concentration of the cross-linker led to a less hydrophobic membrane and higher degree of acrylate conversion. However, a minimum of 2 wt% cross-linker was required to obtain sufficient cross-linking and improved solvent stability. Moreover, a higher energy dose resulted in a higher cross-linking degree but generated more heat at the membrane surface, which led to a significant loss of surface porosity. When the energy density was halved, the permeance of the UV-cured PSf supports increased drastically from 22.5 to 406.3 L m^−2^ h^−1^ bar^−1^. PA TFC membranes with promising separation performance (98% RB rejection and water permeance up to 1.5 L m^−2^ h^−1^ bar^−1^) were successfully synthesized, which demonstrated the potential of UV-cured PSf membranes as solvent-resistant supports for the preparation of TFC membranes.

## Figures and Tables

**Table 1:** Composition of the reference (M0) and UV-cured PSf membranes (M1–M4).

	M0	M1	M2	M3	M4
PSf type	3010 or 6010	3010	3010	6010	6010
PSf (wt%)	10	10	10	10	10
Tamisolve (wt%)	90	84.7	84	84.7	84
XL (wt%)	-	5	5	5	5
PhIn (wt%)	-	0.3	1	0.3	1

**Table 2:** Qualitative observation of the solvent stability of the reference membrane (M0) and UV-cured PSf membranes (M1–M4).

	M0	M1	M2	M3	M4
Ethyl acetate	X	1	1	1	1
NMP	0	X	X	X	X
THF	0	X	X	X	X
Toluene	0	1	1	1	1

0: Dissolving, X: swelling, 1: stable.

**Table 3:** Qualitative observation of the solvent stability of UV-cured PSf supports prepared with different cross-linker concentrations.

XL (wt%)	0	0.5	1	1.5	2
Ethyl acetate	X	X	X	X	1
NMP	0	0	0	0	X
THF	0	0	0	0	X
Toluene	0	0	0	0	1

**Table 4:** Qualitative observation of the solvent stability of UV-cured PSf supports consisting of 10, 12, or 14 wt% PSf, 0.3 wt% PhIn, and varying cross-linker concentrations.

XL (wt%)	0	2	3	4
Ethyl acetate	X	1	1	1
NMP	0	X	X	X
THF	0	X	X	X
Toluene	0	1	1	1

## Data Availability

Not applicable.
